# Asthma Symptom Self-Monitoring Methods for Children and Adolescents: Present and Future

**DOI:** 10.3390/children12080997

**Published:** 2025-07-29

**Authors:** Hyekyun Rhee, Nattasit Katchamat

**Affiliations:** 1 School of Nursing, University of Texas at Austin, 1710 Red River St., Austin, TX 78712, USA; nk24573@my.utexas.edu; 2 Ramathibodi School of Nursing, Faculty of Medicine Ramathibodi Hospital, Mahidol University, 270, Rama 6, Rachathewi, Bangkok 10400, Thailand

**Keywords:** asthma, children, adolescents, symptom monitoring, symptom-based, peak expiratory flow rate, symptom quantification, sensor-based monitoring, wearables

## Abstract

Asthma is the leading chronic condition in children and adolescents, requiring continuous monitoring to effectively prevent and manage symptoms. Symptom monitoring can guide timely and effective self-management actions by children and their parents and inform treatment decisions by healthcare providers. This paper examines two conventional monitoring methods, including symptom-based and peak expiratory flow (PEF) monitoring, reviews early efforts to quantify respiratory symptoms, and introduces an emerging sensor-based mHealth approach. Although symptom-based monitoring is commonly used in clinical practice, its adequacy is a concern due to its subjective nature, as it primarily relies on individual perception. PEF monitoring, while objective, has shown weak correlations with actual asthma activity or lung function and suffers from suboptimal adherence among youth. To enhance objectivity in symptom monitoring, earlier efforts focused on quantifying respiratory symptoms by harnessing mechanical equipment. However, the practicality of these methods for daily use is limited due to the equipment’s bulkiness and the time- and labor-intensive nature of data processing and interpretation. As an innovative alternative, sensor-based mHealth devices have emerged to provide automatic, objective, and continuous monitoring of respiratory symptoms. These wearable technologies offer promising potential to overcome the issues of perceptual inaccuracy and poor adherence associated with conventional methods. However, many of these devices are still in developmental or testing phases, with limited data on their clinical efficacy, usability, and long-term impact on self-management behaviors. Future research and robust clinical trials are warranted to establish their role in asthma monitoring and management and improving asthma outcomes in children and adolescents.

## 1. Introduction

Asthma is a pervasive respiratory condition that affects people across all age groups worldwide. It is particularly prevalent among children, with an estimated 81 million affected globally [1], and is associated with greater morbidity and mortality in youth compared to adults [2]. In the United States (USA), 6.5% of children under 18 years (approximately 4.7 million) have current asthma. Of those, nearly 40% of children reported experiencing one or more asthma attacks in the previous year [3]. Asthma is a leading cause of school absenteeism, accounting for nearly 14 million missed school days annually [4], and contributes substantially to the economic burden of pediatric healthcare [5,6]. The condition also negatively affects quality of life for both children and parents [7,8,9], with over half of children with asthma reporting some degree of activity limitations [7,10]. Despite the availability of effective treatments to prevent and reduce symptoms [11], achieving optimum asthma control remains elusive. Inadequate symptom monitoring and management are common [12,13,14], leading to suboptimal asthma outcomes and increasing the risk of long-term lung damage. Given the high prevalence, significant morbidity, economic costs, and potential for lasting health consequences and well-being, there is an urgent need for effective strategies to improve pediatric asthma outcomes.

Self-management is a key determinant of asthma outcomes [15,16,17]. It encompasses a range of actions individuals take to prevent, monitor, manage, and communicate symptoms to mitigate the untoward effects of the disease [18]. Establishing and maintaining routines such as symptom monitoring and treatment adherence are central to effective asthma self-management. Of many actions, symptom monitoring is considered foundational, as heightened symptom awareness enables an array of management actions in a timely manner, including taking or adjusting medications, modifying activity levels, avoiding environmental triggers, and seeking medical assistance when needed. Furthermore, monitored symptoms provide valuable critical information that supports healthcare providers in phenotyping the disease, selecting appropriate treatments, assessing treatment response, and predicting future outcomes.

A Cochrane review revealed that children who appropriately monitor their asthma experience fewer cases of asthma exacerbation, make fewer acute care visits, and report better functional outcomes and higher quality of life [19]. Moreover, greater accuracy in symptom perception is associated with reduced school absenteeism and fewer emergency department visits, even after accounting for underlying asthma severity [20,21,22]. Given this evidence, national guidelines for asthma management emphasize ongoing patient-initiated asthma monitoring by tracking either symptoms or peak expiratory flow rates [15]. In addition, in responding to this need, several innovative solutions have emerged to automate symptom monitoring.

Several reviews have been conducted on asthma monitoring in clinical settings [23,24,25,26] and digital home monitoring methods [27,28,29,30,31,32]. However, no reviews have focused on patient-centered approaches for asthma self-monitoring among children and adolescents, with an emphasis on the advantages and challenges of each method. Although there is one review that examines patient-centered monitoring approaches [33], its scope is limited to conventional methods, including only symptom-based and peak expiratory flow monitoring. Thus, this narrative review aims to provide a more comprehensive overview of the currently available asthma self-monitoring approaches, including symptom-based monitoring, peak expiratory flow rate monitoring, and sensor-based symptom quantification methods, with careful examination of challenges associated with each method as well as the opportunities they present for improving disease management.

## 2. Asthma Monitoring Methods

### 2.1. Symptom-Based Monitoring

Symptom-based monitoring remains the most commonly used strategy in current practice. It does not require standardized tools or formal training; rather, patients are typically encouraged to track their symptoms in either a paper or electronic diary and share this information with their providers. However, many studies have raised concerns about the clinical usefulness of patient-reported symptoms, due to questionable perceptual accuracy of symptoms among children and caregivers [17,21,34,35,36,37,38]. Symptom perception relies on awareness, which involves unconscious, often flawed information processing that is highly influenced by circumstances. Indeed, inaccurate symptom perceptions are widespread among children and present a major barrier to effective disease management. As such, it is associated with increased asthma morbidity, greater use of acute care services, and reduced quality of life [20,36,39,40,41,42].

Symptom perception can be biased in two directions: underperception or overperception [43]. Underperception occurs when children perceive themselves as asymptomatic despite experiencing symptoms, whereas overperception refers to perceiving symptoms as more severe than they are or when none are present. Underperception is more commonly reported than overperception among pediatric populations [36,44]. Underperception may lead to denial or downplaying of symptoms, neglecting or delaying care-seeking and resulting in suboptimal treatment. Conversely, overperception can result in unnecessary activity restrictions, overuse of medications, or excessive healthcare utilization [7,36]. Notably, adolescents are predominantly underperceivers [7,21,34,35,45,46]. Three key factors contribute to inadequate symptom perception, particularly underperception.

#### 2.1.1. The Chronic Nature of Asthma

Asthma’s chronicity may lead to symptom habituation and desensitization, responsible for underperception [21,38]. Nearly two-thirds of adolescents with asthma were diagnosed before age six [47], and prolonged exposure to symptoms for many years may lead to adaptation or dismissive attitudes toward them [13,48]. As children habituate, asthma episodes elicit fewer negative emotional responses, further diminishing perceptual accuracy [44]. Similarly, parents become complacent over time, downplaying symptoms in their children or overlooking changes in condition, which delays or prevents adequate and timely treatment [49]. Children often model these parental behaviors or attitudes, further impairing their own symptom awareness [50].

#### 2.1.2. Inability to Detect or Report Nocturnal Symptoms

Poor perception often occurs in association with nighttime symptoms than daytime symptoms due to patients’ inability to recount nocturnal symptoms [21,35,51]. Nocturnal symptoms are common and critical to gauging the degree of asthma control. Asthma-related cough typically increases at the onset of night sleep, within the first 30 min to 1 h in bed [52,53]. Even children with mild asthma and normal spirometry can exhibit high levels of nocturnal wheezing [51]. Asthma symptoms often worsen at nighttime [54]. However, these symptoms often go unrecognized, although they are strong indicators of poorly controlled asthma or imminent asthma attacks [53,55], with significant effects on sleep, daily functioning, academic performance, and behavior [51]. They occur in 47–67% of patients with asthma and are more common in those with longer disease duration [21]. Yet, many asthma patients fail to recognize these symptoms as a sign of worsening asthma and underreport them [56,57,58,59]. Despite their clinical importance, adequately capturing nighttime symptoms remains a major challenge.

#### 2.1.3. Symptom Underperception in Severe Cases of Asthma

Underperception or underestimation of symptoms often occurs in association with higher asthma severity [37]. Indeed, children with increased bronchial responsiveness, persistent airway inflammation, or low baseline FEV1 levels often exhibit diminished or impaired symptom perception [60,61,62]. Kifle et al. [63] found that children with a history of life-threatening asthma demonstrated substantially reduced perceptual sensitivity to increased inspiratory resistive loads. This impaired perception poses serious risks, as failure to recognize worsening symptoms can lead to deteriorating and life-threatening asthma attacks or even death [50,63,64,65,66].

As such, there are inherent limitations of symptom-based monitoring, which relies on subjective perception influenced by a range of individual and disease factors as described. This underscores a need for alternative or supplementary methods, such as peak flow monitoring, that offer more objective measures of asthma control.

### 2.2. Peak Expiratory Flow Rate (PEFR) Monitoring

Peak expiratory flow rate (PEFR) monitoring is often recommended to introduce objectivity into asthma monitoring, particularly for individuals with more severe asthma due to their increased likelihood of diminished symptom perception, as discussed earlier. PEFR monitoring is often integrated into an asthma action plan and self-management, leading to improved medication adherence and asthma control, and reduced acute healthcare utilization and school absenteeism [67,68,69]. However, the value of PEFR has been questioned as it fails to objectively represent lung function [70,71] and its poor reflection of changes in asthma conditions [72,73,74]. Moreover, because PEFR is a late indicator of deteriorating asthma control, emerging with the onset of symptoms, it lacks the capacity to predict future asthma activity [75]. While some studies report the positive effects of PEFR monitoring on asthma outcomes in pediatric patients [76,77,78,79], including improved confidence in asthma self-management among children [80], many studies have failed to demonstrate the superiority of PEFR monitoring over symptom-based monitoring [74,76,81,82,83,84,85]. Hence, the effectiveness of PEFR monitoring may vary depending on the reliability of PEFR values and their proper use.

Ensuring the reliability of PEFR values remains an ongoing challenge, as measurement can vary depending on the device used and the user’s technique [86]. Furthermore, peak flow values often show a strikingly weak or no correlation with other key indicators of disease activity, such as forced expiratory volume in one second (FEV1), bronchodilator responsiveness, asthma severity scores, and quality of life [73,87]. In children and adolescents, peak flow monitoring is particularly challenging due to poor adherence, suboptimal techniques, and the effort-dependent nature of the test. Additional concerns include the questionable long-term sustainability of routine monitoring and the potential for inaccurate or fabricated readings [55,76,85,88,89,90].

Taken together, the inconclusive evidence on clinical benefits, concerns regarding measurement reliability, and challenges in maintaining adherence and data integrity seriously undermine the utility of PEFR monitoring. Moreover, as peak flow readings reflect only a single moment in time, they fail to capture the dynamic, fluctuating nature of the asthma condition across the day and night. Given these shortcomings, peak flow monitoring may pose an unnecessary burden for pediatric patients, particularly in the absence of a strong clinical justification. Therefore, there is a need for alternative methods that address the noted shortcomings of both symptom-based and peak flow monitoring, which are more objective, reliable, user-friendly, and feasible for long-term use.

### 2.3. Early Attempt to Quantify Respiratory Symptoms

Efforts have been made to objectively measure respiratory symptoms. Since airway obstruction produces characteristic respiratory manifestations, most notably audible symptoms such as coughing and wheezing, technologies aimed at capturing and analyzing acoustic signals have emerged as promising tools. These innovations suggest the potential for novel, noninvasive methods of symptom detection, particularly coughing and wheezing.

Coughing is the most common, cardinal symptom of asthma [91], and its presence often signals a moderate to severe type of uncontrolled asthma if chronic [92,93,94,95]. Chronic cough in asthma is linked to lower lung function and increased healthcare utilization [91]. Persistent coughs are particularly prevalent in young people with asthma, often becoming more pronounced before and during exacerbations [96,97]. However, children often struggle to accurately perceive their own coughs, as suggested by discrepancies between self-reported symptoms and objective assessments [98]. This challenge has driven efforts over the past three decades to develop objective methods for cough detection [99,100,101]. For instance, one notable development is the LR 100 device [101]. This multiparametric recording device, worn in a waist bag, uses a microphone and three electromyographic (EMG) leads attached to the chest. It defines cough by rapid, phasic bursts of signals from the EMG leads, combined with an audio signal from the microphone [101]. Another approach focuses solely on audio signals captured by a tracheal sound recording system, which includes a microphone, transmitter, receiver, and recording equipment [96].

Wheezing is another important symptom of asthma, often signaling poorly controlled asthma and airway obstruction [102]. Research has shown strong negative correlations between wheezing and FEV1 [103,104]. Efforts to quantify wheezing date back to as early as 1984 [105], when researchers used electronic stethoscopes and a cassette recorder to capture brief recordings of wheezing before and after bronchodilator treatment. Since then, the validity and reliability of wheeze sound analysis have been well-supported, establishing it as a valuable marker of airway obstruction and in diagnosing asthma [104,106,107]. More recently, Fiz et al. [104] developed a more advanced technique for recording wheezing using a microphone attached to the skin of the trachea, and the captured sounds were analyzed with a frequency–time algorithm. The value of continuous home recording of wheezing has also been demonstrated using a similar tracheal sound recording system, proving effective in detecting asthma exacerbations in children [107].

Early efforts to quantify respiratory symptoms were primarily conducted in a laboratory for short monitoring periods. When applied in an ambulatory setting, the monitoring equipment, often involving multiple wires and bulky recording devices, severely restricts patient mobility, limiting its utility for daily use. Moreover, analyzing and interpreting recorded data required highly specialized expertise and relied on complex and labor- and time-intensive procedures, making real-time access to symptom information impossible for patients. Because of the inevitable delay between symptom occurrence and analysis, these early methods of quantification methods had limited utility for timely and actionable asthma monitoring. These limitations underscore the need for innovative solutions that enable continuous, objective, real-time asthma monitoring, offering ease of use and direct relevance to asthma self-management and clinical outcomes.

### 2.4. mHealth Approach to Objective Respiratory Symptom Monitoring

Over the past two decades, the application of mobile technologies in health promotion or disease management has grown rapidly, generating the term “mHealth”, broadly defined as “the use of mobile communication and computing technologies in health care and public health” [108]. In 2024, the PeARL (The Pediatric Asthma in Real Life) groups and major professional organizations cautiously endorsed the potential benefits of using mHealth technologies for asthma monitoring between clinical visits [23]. The potential of mHealth as a flexible and scalable alternative to asthma monitoring has been increasingly recognized, particularly for its capacity to collect and track real-time symptom data as they occur in everyday environments. mHealth combined with sensor technology can further automate objective symptom monitoring.

Unlike conventional mobile apps that merely provide a convenient digital platform where users manually record monitored symptoms, sensor-based mobile devices—often in the form of wearables—offer automated, objective, and real-time asthma monitoring. These devices use algorithms to automatically define and detect the acoustic signatures of respiratory symptoms, affording greater accuracy and reliability. Rhee et al. were the first to report on the development and validity of a sensor-based mobile device employing their patented technology to quantify and automate respiratory symptom tracking, along with accelerometer data [109,110,111]. Since then, several wearable sensor systems have been introduced, capable of automatically detecting respiratory symptoms along with other biometrics (e.g., heart rate and respiratory rate), leveraging machine learning algorithms.

In many cases, dedicated sensor devices have been designed to detect respiratory symptoms [111,112,113,114,115,116,117,118], whereas others leverage existing mobile devices such as smartphones [119,120,121,122,123], tablets [124], or smartwatches [125,126] as platforms for symptom algorithms that analyze acoustic information transmitted through their built-in microphones.

Coughing is often the targeted symptom for these wearables [111,113,114,115,127,128,129,130], although few devices are designed to detect wheezing alone [112,116] or both [118]. The reported accuracy of these devices for cough detection is generally high, with sensitivity and specificity ranging from 88.50% to 90.4% and from 99.00% to 99.97%, respectively [125,130,131]. Likewise, high accuracy of detecting wheezing has also been achieved, with high sensitivity and specificity at 98% [131], as shown in Table 1. Many of these devices have been developed for and evaluated in specific diseases exhibiting respiratory symptoms. One device was developed and tested for asthma [111], two were examined in individuals with chronic cough [132,133], and two were tested in patients with COVID-19 [127,134]. Some devices were evaluated in patients with tuberculosis [135], COPD [118], and cystic fibrosis [129], while others have broadly targeted patients with nonspecific respiratory conditions [112,115,120,130,136].

For a symptom monitoring tool, patients must be able to readily access and review monitored symptom data to effectively guide their management actions. Three wearable devices provide an associated smartphone app or device website on which patients can review and track quantified symptoms (e.g., number of coughs) in real time [109,121,129]. Of those, two have been evaluated for usability beyond 24 h as monitoring tools in non-clinical settings [111,129], in which users perceived that these devices were easy to use [109,129] and helpful in understanding and managing their asthma [109].

Quantified coughing and/or wheezing leveraging sensor technologies have successfully demonstrated their clinical value in reflecting current disease status as well as predicting future outcomes. Quantified coughing was associated with unfavorable outcomes among patients with COVID-19 [134], poor treatment response in patients with tuberculosis [135], refractory chronic cough [137], and disease severity and quality of life in children with bronchitis [138]. In asthma, quantified nighttime wheezing was associated with uncontrolled asthma in adults [139]. Similarly, significant associations were reported between cough counts and uncontrolled control, quality of life, and acute healthcare utilization in adolescents with persistent asthma [111]. In addition to concurrent associations, predictive relationships have also been demonstrated between quantified symptoms and future asthma outcomes. For instance, Rhee et al. [111] found that quantified cough data significantly predicted asthma control and quality of life three months later. These findings highlight the potential of sensor-based mobile devices as tools for respiratory symptom monitoring, capable of providing clinically relevant data that inform both current disease conditions and future outcomes.

While sensor-based asthma monitoring offers promising benefits, it may not be practical or accessible for families with low socioeconomic status [140]. These devices often involve high upfront costs, require smartphones or tablets with reliable internet access, and depend on technological literacy that may be limited in under-resourced communities [141]. Therefore, careful consideration of the family’s internet access, affordability, and digital literacy is essential in recommending sensor-based tools for pediatric asthma self-monitoring.

## 3. Limitations

This narrative review has some limitations. First, the articles included were selected based on the alignment of their findings with the focus of this review. Moreover, this review is solely based on articles published in peer-reviewed journals. As a result, selection bias and publication bias may be present, as some studies might have been unintentionally excluded. Second, this review uses a descriptive method to summarize the existing studies, rather than critically synthesizing their findings. Consequently, the methodology and quality of the included studies were not considered, which might increase biases in our review.

## 4. Recommendations for Future Research

To critically evaluate and synthesize the clinical effects of each self-monitoring method for asthma in children and adolescents, a systematic review or meta-analysis is required. Despite the anticipated benefits of sensor-based monitoring approaches, many devices remain in early development or testing stages, with limited data on their validity, usability, or impact on self-management behaviors compared to traditional monitoring methods. Moreover, it remains largely unknown how quantified symptom data translate into improved disease outcomes or quality of life, particularly in pediatric populations. To drive widespread adoption of these innovative tools, rigorous clinical trials need to be conducted to establish their clinical efficacy and cost-effectiveness. In addition, long-term adherence to these devices must be carefully evaluated to determine the real-world sustainability of sensor-based asthma monitoring methods.

## 5. Conclusions

Symptom monitoring is fundamental to asthma self-management, as effective prevention and treatment hinge on patients’ awareness of symptoms. Currently, national asthma guidelines recommend symptom-based and peak flow monitoring. However, the clinical utility of these methods has been questioned due to issues such as poor symptom perception, low adherence, and inadequate skill, as well as their inconclusive capacity to reflect the asthma condition. To overcome the limitations of subjective symptom reporting, early efforts were made to detect and quantify respiratory symptoms using technical equipment. However, these earlier quantification methods were impractical for daily use due to bulky, complex equipment, limited mobility, and labor- and time-intensive data processing, which made real-time feedback impossible.

Recently, rapid advances in sensor and mobile technologies have paved the way for a new era of asthma monitoring through real-time symptom quantification and feedback. Several wearable devices have been developed that automatically detect and track respiratory symptoms. These wearables represent a paradigm shift in asthma monitoring, moving away from subjective perceptions to objective sensors, enabling continuous and effortless symptom tracking throughout the day and night. Moreover, some of these devices offer real-time feedback by conveniently displaying monitored symptoms directly to users. This feature not only increases patient awareness of their symptoms but also helps them understand dynamic play between management behaviors (e.g., medication taking and trigger mitigation) and symptom trajectories, enabling early detection and prevention of symptom exacerbation. However, many of these devices remain in the developmental phase, with limited evidence regarding their validity, usability, and impact on patients’ outcomes. Future research is needed to better understand the clinical and economic value of these advanced, technology-based asthma monitoring tools compared to conventional monitoring methods.

## Figures and Tables

**Table 1:** Accuracy of sensor-based devices in detecting or classifying respiratory symptoms.

Author (Year)	Country	Participants, Sample Size (n), Number of Sound Epochs (n_s_)	Cough or Wheeze	Se	Sp	PPV	NPV	AUC	P	ACC
Rhee et al. (2015) [111]	USA	Children and adolescents (13–17 years) with asthma (n = 84) n_s_ = Not mentioned	Cough	51.3	72.7	-	-	0.71	-	-
Lonini et al. (2021) [113]	USA	Adults (≥21 years) with COVID-19 (n = 14) n_s_ = Not mentioned	Cough	-	-	-	-	0.64	-	-
Ni et al. (2021) [128]	USA	Adults (≥21 years) with COVID-19 (n = 27) n_s_ = 10,285	Cough	87.00	96.00	-	-	0.97	0.85	90.00
Urban et al. (2022) [131]	Germany	Children and adolescents (1–17 years) with and without respiratory conditions (n = 115) n_s_ = 92,976	Cough	89.70	99.70	79.43	99.86	-	-	99.50
			Wheeze	97.60	97.90	71.03	99.87	-	-	97.90
Kuhn et al. (2023) [130]	Switzerland	Adults (≥18 years) with respiratory conditions (n = 27) n_s_ = Not mentioned	Cough	Day 88.55 Night 84.24	Day 99.97 Night 99.97	Day 89.68 Night 87.06	Day 89.68 Night 87.06	-	-	-
Sanchez-Morillo et al. (2024) [115]	Spain	Male and female participants without a specified age group (n = 23) n_s_ = 1217	Cough	90.25	94.70	-	-	-	0.95	92.21
Tzavelis et al. (2024) [129]	USA	Children (3–18 years) with cystic fibrosis (n = 50) n_s_ = Not mentioned	Cough	-	-	-	-	0.97	0.96	95.00
Chaccour et al. (2025) [125]	USA	Adults (≥21 years) with respiratory conditions (n = 28) n_s_ = 4454	Cough	90.4	-	87.5	-	-	-	-

Note: Se = sensitivity, Sp = specificity, PPV = positive predictive value, NPV = negative predictive value, AUC = area under the curve, P = precision, ACC = accuracy.

## Data Availability

Not applicable.
